# Predicted Aflatoxin B_1_ Increase in Europe Due to Climate Change: Actions and Reactions at Global Level

**DOI:** 10.3390/toxins13040292

**Published:** 2021-04-20

**Authors:** Marco Camardo Leggieri, Piero Toscano, Paola Battilani

**Affiliations:** 1 Department of Sustainable Crop Production (DI.PRO.VE.S.), Università Cattolica del Sacro Cuore, Via Emilia Parmense 84, 29122 Piacenza, Italy; marco.camardoleggieri@unicatt.it; 2 IBE-CNR, Institute of BioEconomy-National Research Council, Via Giovanni Caproni 8, 50145 Florence, Italy; piero.toscano@ibe.cnr.it

**Keywords:** *Aspergillus flavus*, mycotoxin, crop modeling, predictive model, co-occurrence, food, feed, risk assessment, safety

## Abstract

Climate change (CC) is predicted to increase the risk of aflatoxin (AF) contamination in maize, as highlighted by a project supported by EFSA in 2009. We performed a comprehensive literature search using the Scopus search engine to extract peer-reviewed studies citing this study. A total of 224 papers were identified after step *I* filtering (187 + 37), while step *II* filtering identified 25 of these papers for quantitative analysis. The unselected papers (199) were categorized as “actions” because they provided a sounding board for the expected impact of CC on AFB_1_ contamination, without adding new data on the topic. The remaining papers were considered as “reactions” of the scientific community because they went a step further in their data and ideas. Interesting statements taken from the “reactions” could be summarized with the following keywords: Chain and multi-actor approach, intersectoral and multidisciplinary, resilience, human and animal health, and global vision. In addition, fields meriting increased research efforts were summarized as the improvement of predictive modeling; extension to different crops and geographic areas; and the impact of CC on fungi and mycotoxin co-occurrence, both in crops and their value chains, up to consumers.

## 1. Conceptual Framework

The mycotoxins of greatest concern to food and feed safety are produced by members of a few genera of filamentous fungi, with *Aspergillus*, *Fusarium* and *Penicillium* playing a key role. These fungi colonize many crops and are adapted to a wide range of environmental conditions, having different but partially overlapping ecological niches [1]. A key point of interest in relation to maize are the aflatoxin (AF) producers *Aspergillus flavus* and *A. parasiticus*, *Fusarium verticillioides* and *F. proliferatum*, known for fumonisin (FB) production, and *F. graminearum*, able to biosynthesize both trichothecenes, such as deoxynivalenol (DON), and zearalenones (ZEN) [2,3]. Among staple crops, maize is of concern for mycotoxin contamination; mycotoxins are regulated in Europe and in several other countries worldwide, and several co-occurring fungal organisms are often detected [4]. 

Knowledge of environmental factors affecting fungal survival, growth, metabolic activity and interaction with other organisms, including host plants, is essential for understanding their dynamics and the resulting toxin contamination [5]. The environment provides all the leading factors for mycotoxin prevalence. In particular, high temperatures and drought stress directly affect maize and the occurrence of *A. flavus*, favoring fungal growth, conidiation and spore dispersal, and impairing the growth and development of maize [6]. FB-producing fungi can be found wherever maize is grown, but their occurrence varies geographically. FB occurrence is typically higher in maize-growing areas at low latitudes and elevations, where conditions are relatively warmer compared with those of high-latitude or high-altitude maize-growing regions where [7,8,9], on the contrary, DON is commonly dominant [2].

Climate change (CC) is predicted to have a significant impact on the security of staple commodities. Based on available data, atmospheric concentrations of CO_2_ are expected to double or triple (from 350–400 to 800–1200 ppb) in the next 25–50 years. Therefore, different regions of Europe is expected to face increases in temperature of 2–5 °C coupled with elevated CO_2_ (800–1200 ppm) and drought episodes, with concomitant effects on pests and diseases and ultimately crop yield [10,11,12], as well as mycotoxins. Until a few years ago, AFs had not been identified as a matter of concern for primary production in Europe. However, the year 2003 saw the first alarming contamination of maize in Italy [13]. AFs are potent carcinogens existing as four primary structural analogues: AFB_1_, AFB_2_, AFG_1_ and AFG_2_. The International Agency for Research on Cancer (IARC) has classified AFB_1_ as a Group 1A carcinogen, i.e., carcinogenic to humans [14]. In addition to hepatocellular carcinoma, AFs are associated with occasional outbreaks of acute aflatoxicoses, leading to death shortly after exposure [15].

The European Food Safety Authority (EFSA), with a mandate to identify emerging risks in the food and feed sectors, has identified changing patterns in mycotoxin production in cereals due to CC as a potential matter of concern. Therefore, in 2009, the EFSA’s Emerging Risks Unit delivered a call for scientific information (CFP/EFSA/EMRISK/2009/01), based on models and scenarios, to predict the possible increase of AFs in cereals in the EU due to CC. Two CC scenarios, +2 °C and +5 °C above pre-industrial levels, which consider whether or not mitigation strategies for CC are applied, in addition to the present (baseline) scenario were considered in the funded project, MODMAP-AFLA. These scenarios provided the data input for AFLA-maize [16], a mechanistic model, able to predict AF contamination risk using weather data as input. The project’s output predicted an increased risk of AF contamination in maize in the future [17,18]. Findings also suggested that CC effects will be (*a*) regional; and (*b*) detrimental or advantageous depending on geographical region and the CC scenario considered [18]. In northern Europe, the effects may be positive, with the enlargement of maize growing area without or with minimal AF risk. Conversely, the Mediterranean basin is expected to be a hot spot of many adverse effects, with extreme changes in rainfall/drought, elevated temperatures and CO_2_ impacting food production and AF contamination in maize.

In this study, we identified the actions and reactions of the scientific community based on the results of the MODMAP-AFLA project [17,18].

### 1.1. Dataset Creation: Scientific Paper Search, Filtering, and Selection

A comprehensive literature search was performed using the Scopus search engine to extract peer-reviewed studies that were published until the end of 2020 (Scopus last access 28 March 2021). The citations included, either (*a*) the EFSA report: Modelling, predicting and mapping the emergence of aflatoxins in cereals in the EU due to climate change [17]; or (*b*) the accompanying manuscript: AFB_1_ contamination in maize in Europe increases due to CC [18]. 

**Figure 1:**
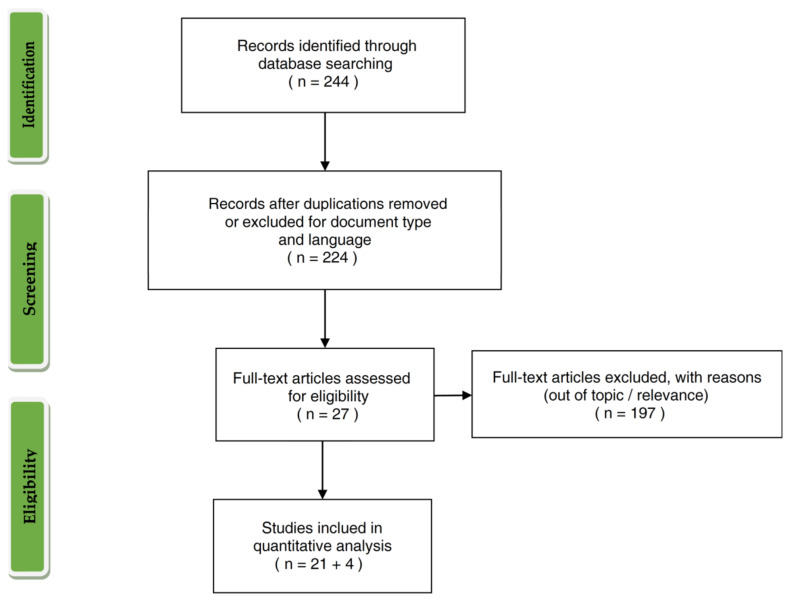
Workflow showing the phases of paper selection.

Two-step filtering was conducted during database creation. The step *I* exploited the exclusion criteria available directly in the Scopus search engine: Document type, and language (Figure 1). Only papers, conference papers, and book chapters published in English were selected.

Bibliometric metadata for the selected research papers were then exported from the Scopus search engine. Metadata text files were elaborated using the scientific mapping software VOSviewer [19].

### 1.2. Topic Categorization and Other Classification Criteria

A second level of filtering was performed to determine eligibility of the selected research papers, based on the following exclusion criteria: (*a*) Adequacy of the paper topic to match the objectives of aflatoxin and CC; (*b*) mixed criterion accounting for at least one topic within (*a*) crop model, (*b*) fungal model, (*c*) weather data, (*d*) climate data, (*e*) current impact, (*f*) future impact and (*g*) single occurrence or co-occurrence (Table 1). For all papers compliant with at least one of the aforementioned criteria, the authors extracted information about the area of study and matrix. The authors then proceeded with a careful reading of the full text of each eligible article.

## 2. Motivations Underpinning Action-Reaction Analysis

This review considers all papers citing the output of EFSA project MODMAP-AFLA [17] on the effect of CC on *A. flavus* and AF contamination in maize across Europe [18]. 

Step *I* filtering identified 224 papers [5,6,20,21,22,23,24,25,26,27,28,29,30,31,32,33,34,35,36,37,38,39,40,41,42,43,44,45,46,47,48,49,50,51,52,53,54,55,56,57,58,59,60,61,62,63,64,65,66,67,68,69,70,71,72,73,74,75,76,77,78,79,80,81,82,83,84,85,86,87,88,89,90,91,92,93,94,95,96,97,98,99,100,101,102,103,104,105,106,107,108,109,110,111,112,113,114,115,116,117,118,119,120,121,122,123,124,125,126,127,128,129,130,131,132,133,134,135,136,137,138,139,140,141,142,143,144,145,146,147,148,149,150,151,152,153,154,155,156,157,158,159,160,161,162,163,164,165,166,167,168,169,170,171,172,173,174,175,176,177,178,179,180,181,182,183,184,185,186,187,188,189,190,191,192,193,194,195,196,197,198,199,200,201,202,203,204,205,206,207,208,209,210,211,212,213,214,215,216,217,218,219,220,221,222,223,224,225,226,227,228,229,230,231,232,233,234,235,236,237,238,239,240,241,242]: 187 citing Battilani, et al. [18] and 37 citing Battilani, et al. [17]. Step *II* filtering identified 25 papers (Table 1; 21 citing [18] and 4 citing [17]) relevant to the study, which were included in a deeper analysis. These papers were categorized as “*reactions*” to the cited results because they went a step further. All the other papers (199) were considered “*actions*” following those publications; they played the role of sounding board for the expected impact of CC on AFB_1_ contamination, without adding new data on the topic.

The overall workflow of database creation with single steps and corresponding number of selected or excluded papers is shown in Figure 1.

## 3. Overview of Selected Papers

**Figure 2:**
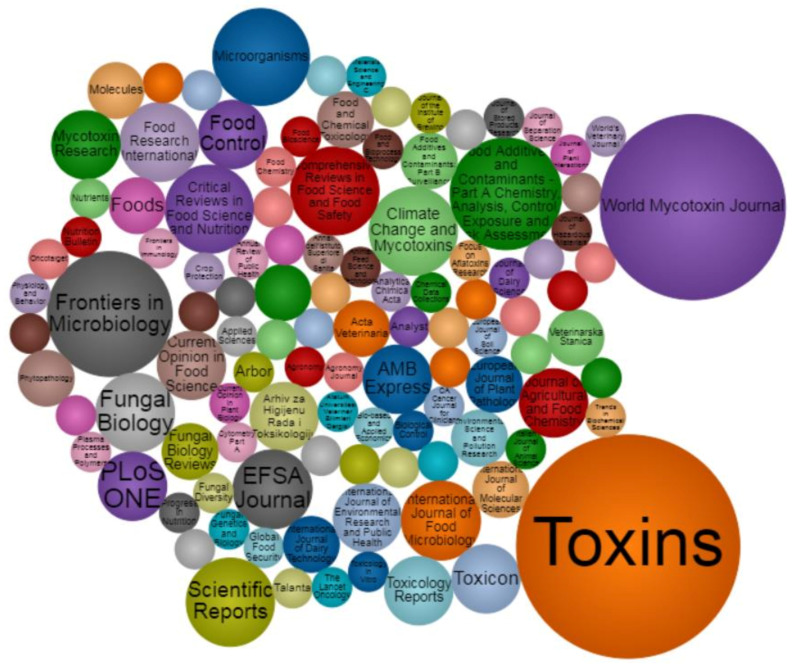
Treemap of all source titles for the records (paper and report citations) identified during step *I* filtering. Treemap elaborated and created using the DrasticData online tool [243].

**Figure 3:**
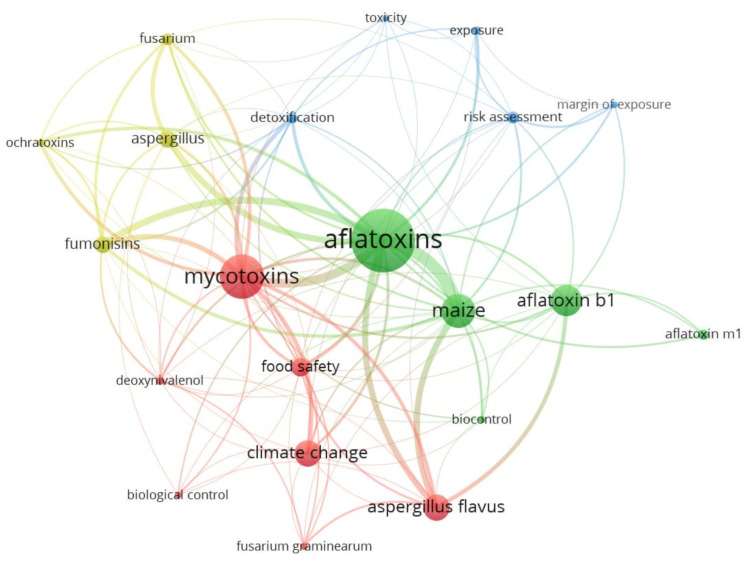
Scientific mapping of all keyword networks based on records (paper and report citations) from step *I* filtering.

**Figure 4:**
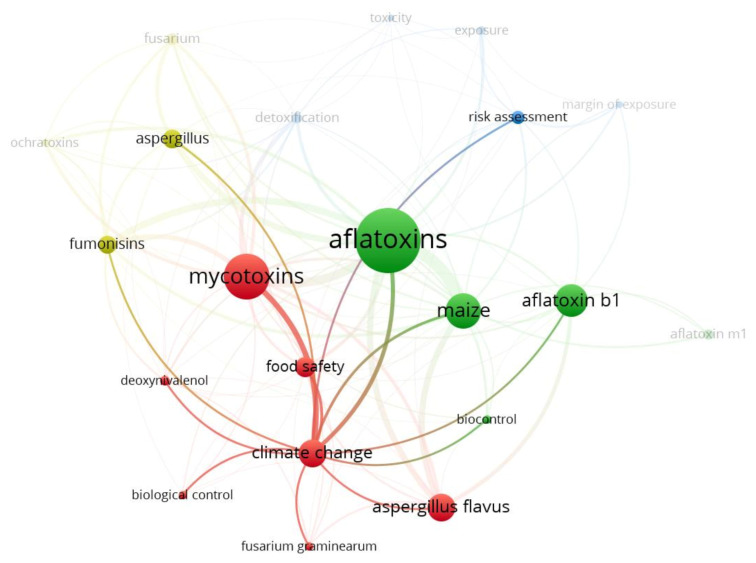
Scientific mapping of strictly linked networks for climate change as keyword, based on records (paper and report citations) from step *I* filtering.

**Figure 5:**
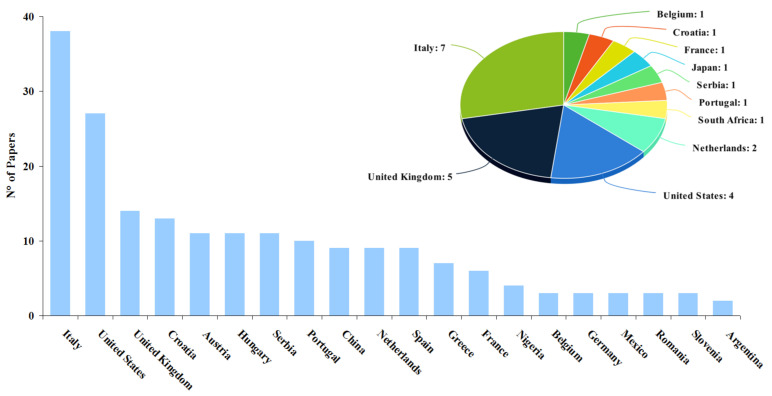
Bar graph showing the top 20 countries affiliated with authors of records from step *I* filtering. [Others: 3 papers each from Belgium, Germany, Mexico, Romania, Slovenia; 2 papers each from Argentina, Canada, India, Iran, Malawi, Malaysia, Philippines, Poland, South Africa, Switzerland, Thailand, Turkey; 1 paper each from Algeria, Brazil, Cyprus, Egypt, El Salvador, Ghana, Haiti, Indonesia, Ireland, Japan, Lithuania, North Macedonia, Pakistan, Saudi Arabia]. Pie chart (upper corner right) refers to the authors’ countries for the 25 studies selected for quantitative analysis.

The results of the scientific mapping, including papers categorized as “*actions*” and “*reactions*,” are summarized in four figures highlighting the journal where papers were published, keywords and their link to each other, and the countries to which the authors were affiliated (Figure 2, Figure 3, Figure 4 and Figure 5).

The source titles for all research papers filtered through the exclusion criteria during the screening process (step *I*—224 papers) are shown in Figure 2. *Toxins* (MDPI) turned out to be, by far, the most popular journal for publication, accounting for 14.3% (32 papers) of the filtered publications, followed by *World Mycotoxin Journal* (9.8%, 22 papers—Wageningen Academic Publishers), *Frontiers in Microbiology* (4.5%. 10 papers—Frontiers Media), *Food Additives and Contaminants—Part A Chemistry*, *Analysis*, *Control*, *Exposure and Risk Assessment* (3.6%, 8 papers—Taylor & Francis Online) and *Microorganism* (2.7%, 6 papers—MDPI).

Despite most of the selected articles (89%, 199 papers) citing Battilani, et al. [17] and Battilani, et al. [18] only in the introduction, or not providing substantial advances to the topic covered by these two publications, our keywords occurrence analysis (Figure 3 and Figure 4) resulted in a well-defined pattern clustering the keywords into four groups, with colored lines indicating strong co-occurrence links between them. In the network mapping shown in Figure 3, (*a*) the first cluster (red color) comprises the keywords “*Aspergillus flavus*,” “biological control,” “climate change,” “deoxynivalenol,” “food safety,” “*Fusarium graminearum*” and “mycotoxins”; (*b*) the second cluster (green color), includes “aflatoxin B1,” “aflatoxin M1,” “aflatoxins,” “biocontrol” and “maize”; (*c*) the third cluster (light blue color) encompasses “detoxification,” “exposure,” “margin of exposure,” “risk assessment” and “toxicity”; while (*d*) the fourth cluster covers “*Aspergillus*,” “fumonisins,” “*Fusarium*” and “ochratoxins.” An in-depth analysis of the co-occurrence of keywords from different clusters (Figure 4) revealed “climate change” as the key element for most papers, with this keyword strongly linked (thick lines) to most of the main keywords of other clusters such as “fumonisins,” “*Aspergillus*,” “aflatoxins,” “maize,” “aflatoxin B_1_” and “risk assessment.” 

The bar graph in Figure 5 displays the top 20 countries affiliated with authors of the selected papers. Italy and the United States were the leading countries where researchers citing Battilani, et al. [17] and Battilani, et al. [18] came from, with 38 and 27 papers, respectively. There were also scientists from the United Kingdom (14), Croatia (13) and Austria (11) together with Hungary and Serbia. This top 20 highlight a deficit of papers from some continents where mycotoxin contamination is considered a major problem, with implications that affect human and animal health (i.e., Africa and Asia). Indeed, only Nigeria (4 papers) and China (9 papers) ranked in this top 20 list. The pie chart (Figure 5—upper corner right) illustrates the authors’ countries for the 25 studies selected for quantitative analysis, considered as “reactions”: Here also, Italy (7), the United Kingdom (5) and the United States (4) were the countries with the largest number of articles.

## 4. Reactions

We selected 25 papers from the final dataset, accounting for the scientific community’s reactions to the topic (Table 1). The eligible research studies were tabulated, according to study area, matrix, model approach, weather data, climate scenario, current and future impact, and mycotoxin occurrence and co-occurrence, in order to highlight the availability of data and to outline some statements based on the above-mentioned tabulating criteria. Most of the matrices analyzed were related to both food and feed (general), while maize was the most represented crop. Milk and dairy products were also present, as well as coffee, tomato, grapes and wheat. The majority (64%) of studies did not implement any models, such as climate models, plant phenology or algorithms, or just referred to the results published in other studies. As expected, most of the work was focused on AFs (AFB_1_, AFM_1_ and total AFs), while their co-occurrence with other mycotoxins (FBs and DON) in the same matrix was only considered in two cases. The analysis of the impact of current climate conditions on mycotoxin contamination was limited to six studies, which was further reduced to three studies if the assessment of the impact of future climate scenarios was also studied.

### 4.1. CC Impact on Aspergillus flavus and Aflatoxin Contamination

First confirmations of the predicted increase in risk of AFB_1_ occurrence in maize under CC scenarios arrived soon after publication of the MODMAP-AFLA report in 2012 [17], with an event occurring in Serbia in the same year [244,245]. This was also the case for France, where, in 2015, exceptionally hot and dry climatic conditions caused 6% of maize fields to be contaminated by aflatoxins. Strains of *Aspergillus* section *Flavi* were isolated from maize samples, and *A. flavus* was the prevalent species (69% of strains), confirming the presence of these potent toxin-producers in fields in France [31], in addition to those reported in Italy before [13,246] and after publication of the report [247].

The same approach reported in the reference papers [17,18] was used effectively to study the outcome of CC on *A. flavus* in maize in Malawi [248]. Malawi is projected to get warmer (by 1–2.5 °C) and drier (reduction of 0–4% in annual rainfall levels) in all regions, with some uncertainty regarding precipitation. These conditions are expected to shorten the maize growing season, with a major impact on long-development varieties, causing the pre-harvest conditions for Malawian maize to become more favorable for AFB_1_ contamination. This was the only study that considered all components of CC, with particular regards to the effect of climate on maize crop phenology, *A. flavus* ecology and expected AFB_1_ contamination of grain. 

The effect of CC was also reviewed in the context of mycotoxigenic fungi in coffee cultivation regions, Mesoamerica and central Africa in particular [21]. CC is expected to modulate the prevalence of fungal species, with a decline in *Penicillium* species and an increase in aflatoxin-producing *Aspergilli* species. In addition, the impact on OTA production seems species dependent. In fact, only for *A. westerdijkiae*, high CO_2_ (1000 ppm), high temperature (30–35 °C) and sub-optimal a_w_ (0.90, 0.95 and 0.97), significantly stimulated OTA production in coffee beans. Suitable coffee growing areas will be affected by CC as well. Predictions suggest that suitable coffee cultivation areas could decrease by ~50% by 2050, both for Arabica and Robusta varieties. All indications showed that CC will have an extremely negative effect on future coffee production worldwide, in terms of both loss of cultivation areas and increase in mycotoxin contamination. In particular, suitable areas will migrate to higher altitudes where temperatures are cooler. Generally, Arabica is expected to fare worse than Robusta. However, more research is needed to understand how shifts in suitable areas for Arabica and Robusta will impact fungi and their mycotoxins under various CC scenarios.

An interesting approach evaluated grain contamination and considered the impact of CC on the maize-milk chain. This case study was based on maize grown in eastern Europe and imported to the Netherlands to be fed—as part of compound feed—to dairy cows. Three different climate models, one AFB_1_ prediction model and five different carryover models (carryover intended as the passage from AFB_1_ in the feed to AFM_1_, its hydroxylated metabolite, in the milk) were used and combined to obtain a predictive tool based on Monte Carlo simulations [191]. The results showed that, given the case study and the scenarios and models used, AFM_1_ contamination in milk is expected to be comparable or to increase in future climates. The outputs were sometimes in disagreement, depending on the model used; nevertheless, this study merits attention for the chain approach suggested. 

The exposure of Serbia’s adult population to AFM_1_ from milk and dairy product consumption in 2015–2018 was examined by Djekic, et al. [64] and confirmed the previous data. In fact, these authors showed a moderate exposure risk compared with similarly managed studies worldwide, but the research underlined the importance of promoting continuous monitoring of feed and dairy supply chains and providing exposure assessment updates, with the exposure variable depending on the monitoring year.

However, all the studies mentioned above were missing essential aspects of fungal and plant interaction. Medina, et al. [128] stressed this critical aspect, underlining the importance of ecological studies to assess how fungal resilience is affected by interacting CC factors. Camardo Leggieri, et al. [45] recently confirmed this concern by using maize grown in 2014 in northern Italy as a case study. Wide unevenness in mycotoxin occurrence was noticed, even within a small area, with changes in the prevalent compound and in the level of contamination. This variability was attributed to CC effects on fungal complex interaction, with the dominant fungal species alternating during the growing season.

The challenging topic of defining the impact of fungal co-occurrence under different meteorological/ecological conditions on mycotoxin contamination was addressed by Giorni, et al. [249], and Camardo Leggieri, et al. [44], respectively, in field and in vitro. *A. flavus*, *F. verticillioides* and *F. graminearum* were artificially inoculated on maize grown in northern Italy in the two-year period 2016–2017. In parallel, *A. flavus* and *F. verticillioides* were inoculated on cornmeal medium and incubated under a wide range of temperature and water activity (a_w_) conditions. Therefore, fungal interactions could be observed under natural conditions, but the impact of temperature and a_w_ could also be studied in detail and modeled. Under natural conditions, AFB_1_ accumulation was stimulated by the presence of *F. graminearum*, while no effects on FBs or DON, caused by *F. verticillioides—F. graminearum* co-occurrence were noticed. Interestingly, the co-occurrence of *A. flavus* with *F. verticillioides* or *F. graminearum* significantly reduced both FBs and DON production. Only *A. flavus* and *F. verticillioides* were included in the in vitro study, and each fungus was affected by the co-occurrence of the other; in particular, showing a decrease in colony diameter of 10%, and 44%, respectively, when they were grown together compared with growth alone. On the contrary, the dynamics of toxin production under different temperature regimes followed a similar trend for fungi grown alone, or together, but with a decrease in production rate and a shift in optimal temperature for AFB_1_ production. Although these preliminary results seem in partial disagreement, they need attention and careful elaboration. They provide basic knowledge for inclusion in predictive models to account for fungi co-occurrence in the CC scenario and to predict resulting mycotoxin co-occurrence. 

Several researchers underlined the importance of acquiring detailed data in vitro on fungal responses to ecological conditions in the context of CC. In particular, Giorni, et al. [211] studied the effect of temperature and relative humidity on *A. flavus* sclerotia sporulation; data obtained were used to develop equations included in the AFLA-maize predictive model [16,204]. 

A step forward in ecological study was explored by Magan and Medina [121]. They examined the relationship between three-way interacting environmental factors, representative of CC scenarios (water stress × temperature + 2/4 °C × elevated CO_2_ 650/1000 ppm) on growth and mycotoxin gene cluster expression for *A. flavus.* This impacted significantly on AFB_1_ production both on maize based medium (around 80 x the control) and on maize grain (x 3–4 the control). Studies on species of the *Aspergillus* section *Circumdati* and *A.* section *Nigri* on maize grain and coffee suggested that, while fungal growth may not be significantly affected, mycotoxin production seems to be stimulated by CC factors, Comparable conclusions were reported by Raiten and Aimone [157], based on ecological studies with a CC perspective on maize grain and coffee. Apart from revealing the up- or down-regulation of genes, a genomic approach represents a powerful tool for exploiting relative toxin production under extreme stress conditions, such as CC scenarios.

Most of the research efforts during recent years have focused on harvest or post-harvest contamination of AFs in feed/food commodities, but the soil ecosystem has been poorly considered. Fouché, et al. [78] recently reviewed studies that addressed the environmental and toxicological consequences of AF contamination, with the aim of clarifying the eventual risk that AF contamination poses to soil ecosystems. Many aspects of AF occurrence, degradation and the effects of its transformation products in the soil environment are still unknown and remain an essential area of research for both soil health and soil productivity. In terms of soil moisture and air temperature changes, a climatic approach is important for future risk assessments of AF contamination.

### 4.2. CC Impact on Other Pathosystems

Following the prediction of CC impact on *A. flavus* and AFB_1_ in maize under CC scenarios, another pathosystem, *Alternaria* spp. in tomatoes and related mycotoxins, was analyzed, this being an emerging matter of concern. Van de Perre, et al. [241] evaluated the effect of CC in two regions, Badajoz in Spain and Krobia in Poland. There was a significant difference in the potential growth of Alternaria among time frame scenarios in Poland, with far future > near future > current time frame. The results suggested that Poland’s situation in the far future (2081–2100) will become similar to Spain’s situation in the present time frame (1981–2000), showing a geographic shift in the problem. There were no significant differences among the scenarios studied for Spain because the higher temperatures predicted will become limiting for *Alternaria* spp. 

Similarly, DON production in wheat was assessed for north-western Europe, indicating that both flowering and complete maturation of wheat will be earlier in the season because of CC effects. At the same time, DON contamination was expected to increase in most of the regions studied, raising initial concentrations by up to three times [242]. *Fusarium* species involved in Fusarium head blight (FHB) of cereals in the CC context were also addressed by Moretti, et al. [131] in 2019. In-depth modifications to the profile of toxigenic *Fusarium* species occurring on kernels at maturity in different global geographical areas are expected. A substantial modification in mycotoxin occurrence profile will most likely cause the advent of new mycotoxin risks in specific regions due to the shift of *Fusarium* species into new environments. 

The CC scenarios examined by Cervini, et al. [48], considering an increase of more than 2.5 times CO_2_ concentration in the northern Apulia region (southern Italy), predicted an increase in colonization rate by *A. carbonarius* and ochratoxin A (OTA) production in grapes, a matter of concern in that Italian region. Furthermore, preliminary evidence indicated that temperature increase, likely to happen in the same area, may reduce both berry spoilage caused by *A. carbonarius* and OTA production in grapes [47]. In particular, with a temperature range 18/31 °C and under water stress conditions (0.93 a_w_), the fungal growth rate was slower than at 0.99 a_w_, but an over-expression of OTA genes was observed. On the contrary, at 20/37 °C a higher growth rate was observed at 0.93 a_w_. Therefore, high T and water stress seem not favorable for OTA production. Predictions of CO_2_ and temperature increase, resulting from CC seem to lead to contrasting results that need to be verified in the future. 

Overall, in the context of ecological studies, only one work [85] addressed the resilience of non-toxigenic strains of *A. flavus* to CC factors to ensure they have the necessary ecological competence to compete effectively and reduce toxin contamination pre- or post-harvest. The efficacy of non-toxigenic strains in controlling AFB_1_ production was supported by expression of target structural and regulatory genes; they maintained biocontrol of AFB_1_ contamination under elevated CC interacting factors (37 °C × 1000 ppm CO_2_ and drought stress).

### 4.3. CC Impact on Human and Animal Health

During recent years, research has focused on studying or reviewing CC impact on fungal behavior and toxin production, as well as on related human health risks. Fanzo, et al. [72] examined the relationships between CC, diets and nutrition through a food system lens. They included food safety issues that were not only focused on mycotoxins, and identified adaptation and mitigation interventions for each step of the food supply chain to move towards a more climate-smart, nutrition-sensitive food system. The authors proposed that climate-smart agriculture is a promising approach for mitigating direct CC constraints. However, more action is needed to link climate-smart approaches to diets and nutrition, especially for the most vulnerable individuals in the population. Hiatt and Beyeler [94] provided a review synopsis of what is known about CC-induced exposure and its relevance for cancer events. Considering the predicted increase in AFs with CC, of etiological importance for liver cancer, no evidence of increases in hepatocellular cancer associated with CC has been directly attributed to AFs.

The food system appears to show good resilience to CC, but this is apparently not the case for livestock, where two specific and possible impacts on the production system were underlined: (i) contamination of livestock feed by mycotoxins; and (ii) animal health under heat stress (HS) conditions [118]. This suggests the importance of linking feed safety with the integrated approach proposed to adequately tackle food safety risks associated with CC, including perspectives from different natural and social sciences [30]. The potential consequences of an incompletely explored perspective of CC must be considered. 

Taking account of the impact of CC as a whole on social and environmental health elements, and of the increased risk of adverse health effects, especially on the most vulnerable groups in the population, such as children and the elderly, the Symposium “Health and Climate Change” was organized in Rome in 2018 as a joint initiative of the Italian Institute of Health and EFSA. The meeting aimed to promote an inter-sectoral and multidisciplinary approach to CC-related events to counteract expected adverse health effects; the launch of the International Charter on Health and Climate was the concrete output [159].

## 5. Steps Forward and Perspectives

On a global level, CC is expected to have significant impacts on plant biogeography and fungal populations, with effects on mycotoxin patterns, as confirmed by predictive approaches and field surveys. AFB_1_ is expected to increase in Europe as a result of CC; this prediction is based on the AFLA-maize model and confirmed by field surveys. This result has captured the scientific community’s attention, as confirmed by the numerous citations gained by the papers reporting this data [17,18]. 

Predictive models have become crucial for addressing future uncertainties and highlighting risk conditions on a geographic basis. They are likely to be essential tools for mycotoxin prediction, in production chain management and as support for all stakeholders, farmers, extension services and policymakers [250,251]. Scientific mapping of keyword networks of papers citing the EFSA project results [17,18] revealed the total absence of “*crop modeling*” as a keyword, although the studies analyzed contemplate most of the topics for a holistic approach. In fact, advances in modeling the impact of CC were very limited, as detailed in “*reactions*”. This is undoubtedly one of the areas where research needs to be encouraged, together with extension to crops other than maize, as pointed out by Van Der Fels-Klerx, et al. [190], as well as other interacting factors, such as insects pests [252]. Furthermore, when evaluating the pressure risk of mycotoxins based on CC, we strongly advise not neglecting a pre-analysis of the suitability of countries/study areas for cultivation and the specific crop for which the current and future impact of mycotoxins must be assessed.

An increased risk of AFs is paired with fungal and related mycotoxin co-occurrence. The modeling approach should therefore include this event. Scarce data is available on this topic, and it is apparently not easy to interpret and convert into quantitative models. Therefore, new efforts should be addressed towards this research field, possibly integrated with the support of omics methodologies.

The top 20 authors’ countries identified Italy, the USA and the UK as leading actors in this area, but surely does not reflect the main countries where AFs are a matter of concern for people’s health, as highlighted very recently [155]. Therefore, major involvement of developing countries in studies aimed at predicting the impact of CC on AF occurrence is strongly desirable. 

Several aspects related to AFB_1_ and CC need more attention, based on our literature review; nevertheless, interesting statements can be captured, which can be summarized using the following keywords: *chain and multi-actor approach*, *intersectoral and multidisciplinary*, *resilience*, *human and animal health*, *global vision*. To further summarize, the food system should be considered as a whole [253], taking advantage of smart agriculture [23]. We can learn from each other, both from different steps in the chain and from different geographic areas. Scenario analyses build on multi-actor, intersectoral and multidisciplinary approaches, which can provide all stakeholders, policymakers and risk managers the best support in facing health threats, related to CC, and build the needed resilience. 

## Figures and Tables

**Table 1:** Overall research paper dataset tabulated according to topic categorization. Reference number refers to bibliography reference; Study area as ISO 3166-1 alpha-2 country code, otherwise Continents or Global for larger study area; a_w_ = water activity; AFB_1_ = aflatoxin B_1_; WOFOST = WOrld FOod STudies; DON = deoxynivalenol; JRC MARS = Joint Research Centre Monitoring Agricultural ResourceS; DAYMET = daily weather observation data; CRONOS = Climate Retrieval and Observations Network Of the Southeast; ECHAM5 = Global climate model 5th generation; HadCM3Q0 = Hadley Centre Coupled Model version 3, A1B Special Report on Emissions Scenarios; HadGEM2-ES = Hadley Centre Global Environment Model version 2 Earth System; RACMO2 = Regional Atmospheric Climate Model version 2; HADRM3Q0 = Hadley Center Regional Model version 3, A1B Special Report on Emissions Scenarios; AFM_1_ = aflatoxin M_1_; OTA = ochratoxin A; AFs = aflatoxins; FBs = fumonisins; NIV = nivalenol; ZEN = zearalenone.

Reference	Study Area	Matrix	Model Approach	Weather Data	Climate Scenario	Current Impact	Future Impact	Mycotoxin Occurrence	Co-Occurrence
**Djekic, et al. [64]**	RS	Milk and dairy products	NO	Speculative	Speculative	2015–2018	NO	AFM1 (AFB_1_ in feed)	NO
**Hiatt and Beyeler [94]**	Global	Speculative	Speculative	Speculative	Speculative	Speculative	Speculative	General	NO
**Adhikari, et al. [21]**	Global	Coffee	Speculative	Speculative	Speculative	Speculative	Speculative	OTA-AFs-FBs	NO
**Fouché, et al. [78]**	Global	Soil/Food/Feed	Speculative	Speculative	Speculative	Speculative	Speculative	AFs	NO
**Cervini, et al. [47]**	IT *	Grape	Water/light/temperature in lab conditions	LAB conditions	Speculative	Speculative	Speculative	OTA	NO
**Camardo Leggieri, et al. [45]**	IT	Maize	aridity index-correlation index	Air temperature, rainfall, relative humidity	Speculative	2014	Speculative	NIV-DON-T2-HT2-ZEN-FBs-AFB1	YES
**Pleadin, et al. [151]**	Europe	Food/Feed	Speculative	Speculative	Speculative	Speculative	Speculative	AFB_1_-OTA-FBs-PATULINE-DON	NO
**Gasperini, et al. [85]**	BR/MX **	Maize	Pre/post harvest + interactions of Air temperature × CO_2_ × a_w_	LAB conditions	Speculative	Speculative	Speculative	AFB_1_	NO
**Van der Fels-Klerx, et al. [191]**	NL/UA	Maize feed in UA/Milk in NL	3 climate models + AFB1 model + WOFOST+ 5 carryover models	JRC MARS	ECHAM5, HadCM3Q0	2005–2017	2030	AFB_1_-AFM1	NO
**Moretti, et al. [131]**	Europe	Food	Speculative	Speculative	Speculative	Speculative	Speculative	AFs-DON	NO
**Labanca, et al. [118]**	IT	Maize for feed	Speculative	Speculative	Speculative	Speculative	Speculative	AFs	NO
**Ricciardi, et al. [159]**	Global	Food	Speculative	Speculative	Speculative	Speculative	Speculative	General	NO
**Cervini, et al. [48]**	IT	Grape	NO	LAB conditions	NO	Speculative	Speculative	OTA	NO
**Iizumi [99]**	Global	Speculative	Speculative	Speculative	Speculative	Speculative	Speculative	General	NO
**Bailly, et al. [31]**	FR	Maize	Speculative	Speculative	Speculative	Speculative	Speculative	AFB_1_	NO
**Damianidis, et al. [57]**	US	Maize	Logistic regression	Weather stations, DAYMET, CRONOS	NO	Speculative	Speculative	AFs	NO
**Fanzo, et al. [72]**	US	Food/ Feed	Speculative	Speculative	Speculative	Speculative	Speculative	General	NO
**Assunção, et al. [30]**	PT	Dietary exposure	NO	Speculative	Speculative	Speculative	Speculative	AFs	NO
**Medina, et al. [128]**	GB	Food	Speculative	Speculative	Speculative	Speculative	Speculative	General	YES
**Raiten and Aimone [157]**	CA/US	Speculative	Speculative	Speculative	Speculative	Speculative	Speculative	General	NO
**Magan and Medina [121]**	GB	Maize and Coffee	Linear regression	Lab conditions	Speculative	Speculative	Speculative	All mycotoxins	NO
**Van de Perre, et al. [241]**	ES/PL	Tomato	Climate + Alternaria model	Weather stations	HadGEM2-ES	1981–2000	2031–2050 2081–2100	Alternaria	NO
**Giorni, et al. [211]**	GB/IT	Maize	NO	NO	NO	NO	NO	AFs	NO
**Van der Fels-Klerx, et al. [242]**	Europe ***	Wheat	Wheat phenology + Climate + DON model	JRC MARS	RACMO2, HADRM3Q0	1975–1994	2031–2050	DON	NO
**Medina, et al. [226]**	Global	Feed/Food	Data from review + in vitro data	Speculative	Speculative	Speculative	Speculative	All mycotoxins	NO

* Lab/in vitro study reproducing climatic conditions of Apulia region (Italy); ** combination of in situ and in vitro studies; *** refers to north-western Europe.

## Data Availability

Not applicable.

## References

[1] Perrone G., Ferrara M., Medina A., Pascale M., Magan N. (2020). Toxigenic fungi and mycotoxins in a climate change scenario: Ecology, genomics, distribution, prediction and prevention of the risk. Microorganisms.

[2] Logrieco A., Bottalico A., Mule G., Moretti A., Perrone G. (2003). Epidemiology of toxigenic fungi and their associated mycotoxins for some mediterranean crops. Eur. J. Plant Pathol..

[3] Bottalico A. (1998). *Fusarium* disease of cereals: Species complex and related mycotoxin profile in europe. J. Plant Pathol..

[4] Palumbo R., Crisci A., Venâncio A., Cortiñas Abrahantes J., Dorne J.L., Battilani P., Toscano P. (2020). Occurrence and co-occurrence of mycotoxins in cereal-based feed and food. Microorganisms.

[5] Medina Á., González-Jartín J.M., Sainz M.J. (2017). Impact of global warming on mycotoxins. Curr. Opin. Food Sci..

[6] Ojiambo P.S., Battilani P., Cary J.W., Blum B.H., Carbone I. (2018). Cultural and genetic approaches to manage aflatoxin contamination: Recent insights provide opportunities for improved control. Phytopathology.

[7] Bush B.J., Carson M.L., Cubeta M.A., Hagler W.M., Payne G.A. (2004). Infection and fumonisin production by *Fusarium verticillioides* in developing maize kernels. Phytopathology.

[8] Miller J.D. (2001). Factors that affect the occurrence of fumonisin. Environ. Health Perspect..

[9] Wu F., Bhatnagar D., Bui-Klimke T., Carbone I., Hellmich R., Munkvold G., Paul P., Payne G., Takle E. (2011). Climate change impacts on mycotoxin risks in us maize. World Mycotoxin J..

[10] Gregory P.J., Johnson S.N., Newton A.C., Ingram J.S. (2009). Integrating pests and pathogens into the climate change/food security debate. J. Exp. Bot..

[11] Bebber D.P., Ramotowski M.A.T., Gurr S.J. (2013). Crop pests and pathogens move polewards in a warming world. Nat. Clim. Chang..

[12] Bebber D.P., Gurr S.J. (2015). Crop-destroying fungal and oomycete pathogens challenge food security. Fungal Genet. Biol..

[13] Piva G., Battilani P., Pietri A., Barug D., Bhatnagar D., Egmond H.P.V., Kamp J.W.V.D., Osenbruggen W.A.V., Visconti A. (2006). Emerging issues in southern europe: Aflatoxins in italy. The Mycotoxin Factbook. Food & Feed Topics.

[14] IARC, World Health Organization (1993). Iarc monographs on the evaluation of carcinogenic risks to humans. Some Naturally Occurring Substances: Food Items and Constituents, Heterocyclic Aromatic Amines and Mycotoxins.

[15] Azziz-Baumgartner E., Lindblade K., Gieseker K., Rogers H.S., Kieszak S., Njapau H., Schleicher R., McCoy L.F., Misore A., DeCock K. (2005). Case-control study of an acute aflatoxicosis outbreak, Kenya, 2004. Environ. Health Perspect..

[16] Battilani P., Camardo Leggieri M., Rossi V., Giorni P. (2013). Afla-maize, a mechanistic model for *Aspergillus flavus* infection and aflatoxin b_1_ contamination in maize. Comput. Electron. Agric..

[17] Battilani P., Rossi V., Giorni P., Pietri A., Gualla A., Van der Fels-Klerx H.J., Booij C.J.H., Moretti A., Logrieco A., Toscano P. (2012). Modelling, predicting and mapping the emergence of aflatoxins in cereals in the eu due to climate change. EFSA Sci. Tech. Rep.

[18] Battilani P., Toscano P., Van der Fels-Klerx H.J., Moretti A., Camardo Leggieri M., Brera C., Rortais A., Goumperis T., Robinson T. (2016). Aflatoxin b_1_ contamination in maize in europe increases due to climate change. Sci. Rep..

[19] Vosviewer—Visualizing Scientific Landscapes. https://www.vosviewer.com/.

[20] Adegbeye M.J., Reddy P.R.K., Chilaka C.A., Balogun O.B., Elghandour M.M.M.Y., Rivas-Caceres R.R., Salem A.Z.M. (2020). Mycotoxin toxicity and residue in animal products: Prevalence, consumer exposure and reduction strategies—a review. Toxicon.

[21] Adhikari M., Isaac E.L., Paterson R.R.M., Maslin M.A. (2020). A review of potential impacts of climate change on coffee cultivation and mycotoxigenic fungi. Microorganisms.

[22] Agbetiameh D., Ortega-Beltran A., Awuah R.T., Atehnkeng J., Elzein A., Cotty P.J., Bandyopadhyay R. (2020). Field efficacy of two atoxigenic biocontrol products for mitigation of aflatoxin contamination in maize and groundnut in ghana. Biol. Control.

[23] Agrimonti C., Lauro M., Visioli G. (2020). Smart agriculture for food quality: Facing climate change in the 21st century. Crit. Rev. Food Sci. Nutr..

[24] Agriopoulou S., Stamatelopoulou E., Varzakas T. (2020). Advances in occurrence, importance, and mycotoxin control strategies: Prevention and detoxification in foods. Foods.

[25] Ali S., Ejaz S., Anjum M.A., Nawaz A., Ahmad S. (2020). Impact of climate change on postharvest physiology of edible plant products. Plant Ecophysiology and Adaptation Under Climate Change: Mechanisms and Perspectives i: General Consequences and Plant Responses.

[26] Alshannaq A.F., Gibbons J.G., Lee M.-K., Han K.-H., Hong S.-B., Yu J.-H. (2018). Controlling aflatoxin contamination and propagation of *Aspergillus flavus* by a soy-fermenting *Aspergillus oryzae* strain. Sci. Rep..

[27] Antiga L., La Starza S.R., Miccoli C., D’Angeli S., Scala V., Zaccaria M., Shu X., Obrian G., Beccaccioli M., Payne G.A. (2020). *Aspergillus flavus* Exploits Maize Kernels Using an “Orphan” Secondary Metabolite Cluster. Int. J. Mol. Sci..

[28] Arce-López B., Lizarraga E., Vettorazzi A., González-Peñas E. (2020). Human Biomonitoring of Mycotoxins in Blood, Plasma and Serum in Recent Years: A Review. Toxins.

[29] Aristil J., Venturini G., Maddalena G., Toffolatti S.L., Spada A. (2020). Fungal contamination and aflatoxin content of maize, moringa and peanut foods from rural subsistence farms in South Haiti. J. Stored Prod. Res..

[30] Assunção R., Martins C., Viegas S., Viegas C., Jakobsen L.S., Pires S., Alvito P. (2018). Climate change and the health impact of aflatoxins exposure in portugal—An overview. Food Addit. Contam. Part A Chem. Anal. Control Expo. Risk Assess..

[31] Bailly S., El Mahgubi A., Carvajal-Campos A., Lorber S., Puel O., Oswald I.P., Bailly J.D., Orlando B. (2018). Occurrence and identification of *Aspergillus* section *flavi* in the context of the emergence of aflatoxins in french maize. Toxins.

[32] Bandyopadhyay R., Ortega-Beltran A., Akande A., Mutegi C., Atehnkeng J., Kaptoge L., Senghor A., Adhikari B., Cotty P. (2016). Biological control of aflatoxins in Africa: Current status and potential challenges in the face of climate change. World Mycotoxin J..

[33] Barukčić I., Bilandžić N., Markov K., Jakopović K.L., Božanić R. (2018). Reduction in aflatoxin m_1_ concentration during production and storage of selected fermented milks. Int. J. Dairy Technol..

[34] Battilani P. (2016). Recent advances in modeling the risk of mycotoxin contamination in crops. Curr. Opin. Food Sci..

[35] Battilani P., Stroka J., Magan N. (2016). Foreword: Mycotoxins in a changing world. World Mycotoxin J..

[36] Bellingeri A., Cabrera V., Gallo A., Liang D., Masoero F. (2019). A survey of dairy cattle management, crop planning, and forages cost of production in Northern Italy. Ital. J. Anim. Sci..

[37] Bellingeri A., Gallo A., Liang D., Masoero F., Cabrera V. (2020). Development of a linear programming model for the optimal allocation of nutritional resources in a dairy herd. J. Dairy Sci..

[38] Benkerroum N. (2019). Retrospective and Prospective Look at Aflatoxin Research and Development from a Practical Standpoint. Int. J. Environ. Res. Public Health.

[39] Bessaire T., Mujahid C., Mottier P., Desmarchelier A. (2019). Multiple mycotoxins determination in food by lc-ms/ms: An international collaborative study. Toxins.

[40] Braun H., Woitsch L., Hetzer B., Geisen R., Zange B., Schmidt-Heydt M. (2018). Trichoderma harzianum: Inhibition of mycotoxin producing fungi and toxin biosynthesis. Int. J. Food Microbiol..

[41] Caceres I., El Khoury R., Bailly S., Oswald I.P., Puel O., Bailly J.-D. (2017). Piperine inhibits aflatoxin B1 production in *Aspergillus flavus* by modulating fungal oxidative stress response. Fungal Genet. Biol..

[42] Caceres I., Khoury A.A., El Khoury R., Lorber S., Oswald I.P., El Khoury A., Atoui A., Puel O., Bailly J.D. (2020). Aflatoxin biosynthesis and genetic regulation: A review. Toxins.

[43] Caceres I., Snini S.P., Puel O., Mathieu F. (2018). Streptomyces roseolus, A Promising Biocontrol Agent Against *Aspergillus flavus*, the Main Aflatoxin B1 Producer. Toxins.

[44] Leggieri M.C., Giorni P., Pietri A., Battilani P. (2019). *Aspergillus flavus* and *Fusarium verticillioides* Interaction: Modeling the Impact on Mycotoxin Production. Front. Microbiol..

[45] Camardo Leggieri M., Lanubile A., Dall’Asta C., Pietri A., Battilani P. (2020). The impact of seasonal weather variation on mycotoxins: Maize crop in 2014 in northern italy as a case study. World Mycotoxin J..

[46] Çatak J., Yaman M., Uǧur H. (2020). Investigation of aflatoxin levels in chips by hplc using postcolumn uv derivatization system. Prog. Nutr..

[47] Cervini C., Gallo A., Piemontese L., Magistà D., Logrieco A.F., Ferrara M., Solfrizzo M., Perrone G. (2020). Effects of temperature and water activity change on ecophysiology of ochratoxigenic *Aspergillus carbonarius* in field-simulating conditions. Int. J. Food Microbiol..

[48] Cervini C., Verheecke-Vaessen C., Ferrara M., García-Cela E., Magistà D., Medina A., Gallo A., Magan N., Perrone G. (2021). Interacting climate change factors (CO2 and temperature cycles) effects on growth, secondary metabolite gene expression and phenotypic ochratoxin A production by *Aspergillus carbonarius* strains on a grape-based matrix. Fungal Biol..

[49] Chaudhari A.K., Singh V.K., Das S., Deepika, Singh B.K., Dubey N.K. (2020). Antimicrobial, aflatoxin b_1_ inhibitory and lipid oxidation suppressing potential of anethole-based chitosan nanoemulsion as novel preservative for protection of stored maize. Food Bioprocess Technol..

[50] Chulze S.N., Palazzini J.M., Lullien-Pellerin V., Ramirez M.L., Cuniberti M., Magan N. (2020). Fusarium species infection in wheat: Impact on quality and mycotoxin accumulation. Wheat Quality for Improving Processing and Human Health.

[51] Cohen S.P., Leach J.E. (2020). High temperature-induced plant disease susceptibility: More than the sum of its parts. Curr. Opin. Plant Biol..

[52] Cowger C., Brown J.K.M. (2019). Durability of quantitative resistance in crops: Greater than we know?. Annu. Rev. Phytopathol..

[53] Czéh Á., Mézes M., Mandy F., Szőke Z., Nagyéri G., Laufer N., Kőszegi B., Koczka T., Kunsági-Máté S., Lustyik G. (2017). Flow cytometry based rapid duplexed immunoassay for *Fusarium* mycotoxins. Cytom. Part A.

[54] Dallabona C., Pioli M., Spadola G., Orsoni N., Bisceglie F., Lodi T., Pelosi G., Restivo F.M., Degola F. (2019). Sabotage at the Powerhouse? Unraveling the Molecular Target of 2-Isopropylbenzaldehyde Thiosemicarbazone, a Specific Inhibitor of Aflatoxin Biosynthesis and Sclerotia Development in *Aspergillus flavus*, Using Yeast as a Model System. Molecules.

[55] Dall’Asta C., Battilani P. (2016). Fumonisins and their modified forms, a matter of concern in future scenario?. World Mycotoxin J..

[56] Damianidis D., Ortiz B.V., Bowen K.L., Windham G.L., Hoogenboom G., Hagan A., Knappenberger T., Abbas H.K., Scully B.T., Mourtzinis S. (2018). Minimum temperature, rainfall, and agronomic management impacts on corn grain aflatoxin contamination. Agron. J..

[57] Damianidis D., Ortiz B., Windham G., Bowen K., Hoogenboom G., Scully B., Hagan A., Knappenberger T., Woli P., Williams W. (2018). Evaluating a generic drought index as a predictive tool for aflatoxin contamination of corn: From plot to regional level. Crop. Prot..

[58] De Santis B., Debegnach F., Gregori E., Russo S., Marchegiani F., Moracci G., Brera C. (2017). Development of a LC-MS/MS Method for the Multi-Mycotoxin Determination in Composite Cereal-Based Samples. Toxins.

[59] Debegnach F., Brera C., Mazzilli G., Sonego E., Buiarelli F., Ferri F., Rossi P.G., Collini G., De Santis B. (2020). Optimization and validation of a LC-HRMS method for aflatoxins determination in urine samples. Mycotoxin Res..

[60] Dellafiora L., Dall’Asta C. (2016). Masked mycotoxins: An emerging issue that makes renegotiable what is ordinary. Food Chem..

[61] Nieto C.D., Granero A., Garcia D., Nesci A., Barros G., Zon M., Fernández H. (2019). Development of a third-generation biosensor to determine sterigmatocystin mycotoxin: An early warning system to detect aflatoxin B1. Talanta.

[62] Dimitrieska-Stojkovikj E. (2018). Increased Health Impact of Aflatoxins Due to Climate Change: Prospective Risk Management Strategies. J. Food Qual. Hazards Control..

[63] Djaaboub S., Moussaoui A., Meddah B., Gouri S., Benyahia K. (2020). Prevalence of Mycoflora and *Fusarium graminearum* Chemotype DON in Wheat in Bechar Province of South-Western Algeria. Acta Phytopathol. Èntomol. Hung..

[64] Djekic I., Petrovic J., Jovetic M., Redzepovic-Djordjevic A., Stulic M., Lorenzo J.M., Iammarino M., Tomasevic I. (2020). Aflatoxins in Milk and Dairy Products: Occurrence and Exposure Assessment for the Serbian Population. Appl. Sci..

[65] Dong Y., Fan L., Liang J., Wang L., Yuan X., Wang Y., Zhao S. (2020). Risk assessment of mycotoxins in stored maize: Case study of Shandong, China. World Mycotoxin J..

[66] Dowd P.F., Johnson E.T. (2017). Insect damage influences heat and water stress resistance gene expression in field-grown popcorn: Implications in developing crop varieties adapted to climate change. Mitig. Adapt. Strat. Glob. Chang..

[67] Echarri E., Vettorazzi A., Lizarraga E., Arce-López B., González-Peñas E. (2019). Review of the analytical methodologies and occurrence data of aflatoxins in cereals and cereal-based foods in spain. Aflatoxins: Biochemistry, Toxicology, Public Health, Policies and Modern Methods of Analysis.

[68] Elgioushy M.M., Elgaml S.A., El-Adl M.M., Hegazy A.M., Hashish E.A. (2020). Aflatoxicosis in cattle: Clinical findings and biochemical alterations. Environ. Sci. Pollut. Res..

[69] Elzupir A.O., Abdulkhair B.Y. (2020). Health risk from aflatoxins in processed meat products in Riyadh, KSA. Toxicon.

[70] Eskola M., Elliott C.T., HajšLová J., Steiner D., Krska R. (2019). Towards a dietary-exposome assessment of chemicals in food: An update on the chronic health risks for the European consumer. Crit. Rev. Food Sci. Nutr..

[71] Eskola M., Kos G., Elliott C.T., HajšLová J., Mayar S., Krska R. (2020). Worldwide contamination of food-crops with mycotoxins: Validity of the widely cited ‘FAO estimate’ of 25%. Crit. Rev. Food Sci. Nutr..

[72] Fanzo J., Davis C., McLaren R., Choufani J. (2018). The effect of climate change across food systems: Implications for nutrition outcomes. Glob. Food Secur..

[73] Fanzo J., Hood A., Davis C. (2020). Eating our way through the Anthropocene. Physiol. Behav..

[74] Fapohunda S.O., Esan A.O., Anjorin T.S. (2017). Biological control of mycotoxins: An update. World’s Vet. J..

[75] Ferri F., Brera C., De Santis B., Collini G., Crespi E., Debegnach F., Gargano A., Gattei D., Magnani I., Mancuso P. (2020). Association between Urinary Levels of Aflatoxin and Consumption of Food Linked to Maize or Cow Milk or Dairy Products. Int. J. Environ. Res. Public Heal..

[76] Ferri F., Brera C., De Santis B., Fedrizzi G., Bacci T., Bedogni L., Capanni S., Collini G., Crespi E., Debegnach F. (2017). Survey on Urinary Levels of Aflatoxins in Professionally Exposed Workers. Toxins.

[77] Ferrigo D., Mondin M., Scopel C., Maso E.D., Stefenatti M., Raiola A., Causin R. (2019). Effects of a prothioconazole- and tebuconazole-based fungicide on *Aspergillus flavus* development under laboratory and field conditions. Eur. J. Plant Pathol..

[78] Fouché T., Claassens S., Maboeta M. (2020). Aflatoxins in the soil ecosystem: An overview of its occurrence, fate, effects and future perspectives. Mycotoxin Res..

[79] Frumkin H., Haines A. (2019). Global environmental change and noncommunicable disease risks. Annu. Public Health.

[80] Fusco V., Chieffi D., Fanelli F., Logrieco A.F., Cho G., Kabisch J., Böhnlein C., Franz C.M.A.P. (2020). Microbial quality and safety of milk and milk products in the 21st century. Compr. Rev. Food Sci. Food Saf..

[81] Gagiu V. (2018). Triticale crop and contamination with mycotoxins under the influence of climate change—Global study. J. Hyg. Eng. Des..

[82] Gagiu V., Mateescu E., Armeanu I., Dobre A.A., Smeu I., Cucu M.E., Oprea O.A., Iorga E., Belc N. (2018). Post-harvest contamination with mycotoxins in the context of the geographic and agroclimatic conditions in romania. Toxins.

[83] García-Díaz M., Gil-Serna J., Vázquez C., Botia M.N., Patiño B. (2020). A comprehensive study on the occurrence of mycotoxins and their producing fungi during the maize production cycle in Spain. Microorganisms.

[84] García-Díaz M., Patiño B., Vázquez C., Gil-Serna J. (2019). A novel niosome-encapsulated essential oil formulation to prevent *Aspergillus flavus* growth and aflatoxin contamination of maize grains during storage. Toxins.

[85] Gasperini A.M., Rodriguez-Sixtos A., Verheecke-Vaessen C., Garcia-Cela E., Medina A., Magan N. (2019). Resilience of Biocontrol for Aflatoxin Minimization Strategies: Climate Change Abiotic Factors May Affect Control in Non-GM and GM-Maize Cultivars. Front. Microbiol..

[86] Gauthier T., Duarte-Hospital C., Vignard J., Boutet-Robinet E., Sulyok M., Snini S.P., Alassane-Kpembi I., Lippi Y., Puel S., Oswald I.P. (2020). Versicolorin A, a precursor in aflatoxins biosynthesis, is a food contaminant toxic for human intestinal cells. Environ. Int..

[87] Gering E., Incorvaia D., Henriksen R., Wright D., Getty T. (2019). Maladaptation in feral and domesticated animals. Evol. Appl..

[88] Ghadiri S., Spalenza V., Dellafiora L., Badino P., Barbarossa A., Dall’Asta C., Nebbia C., Girolami F. (2019). Modulation of aflatoxin b_1_ cytotoxicity and aflatoxin m_1_ synthesis by natural antioxidants in a bovine mammary epithelial cell line. Toxicol. In Vitro.

[89] Gilbert Sandoval I., Wesseling S., Rietjens I.M.C.M. (2019). Aflatoxin b_1_ in nixtamalized maize in Mexico; occurrence and accompanying risk assessment. Toxicol. Rep..

[90] Girona A.J.R., Sillué S.M., Gahete F.M., Donat P.V., Almenar V.S. (2020). Mycotoxins: The silent enemy. Arbor.

[91] Gömöri C., Nacsa-Farkas E., Kerekes E., Vidács A., Bencsik O., Kocsubé S., Khaled J., Alharbi N., Vágvölgyi C., Krisch J. (2018). Effect of essential oil vapours on aflatoxin production of *Aspergillus parasiticus*. World Mycotoxin J..

[92] Gonçalves A., Gkrillas A., Dorne J.L., Dall’Asta C., Palumbo R., Lima N., Battilani P., Venâncio A., Giorni P. (2019). Pre- and Postharvest Strategies to Minimize Mycotoxin Contamination in the Rice Food Chain. Compr. Rev. Food Sci. Food Saf..

[93] Gruber-Dorninger C., Novak B., Nagl V., Berthiller F. (2017). Emerging mycotoxins: Beyond traditionally determined food contaminants. J. Agric. Food Chem..

[94] Hiatt R.A., Beyeler N. (2020). Cancer and climate change. Lancet Oncol..

[95] Hojnik N., Modic M., Walsh J.L., Žigon D., Javornik U., Plavec J., Žegura B., Filipič M., Cvelbar U. (2021). Unravelling the pathways of air plasma induced aflatoxin B1 degradation and detoxification. J. Hazard. Mater..

[96] Hojnik N., Modic M., Žigon D., Kovač J., Jurov A., Dickenson A., Walsh J.L., Cvelbar U. (2021). Cold atmospheric pressure plasma-assisted removal of aflatoxin B 1 from contaminated corn kernels. Plasma Process. Polym..

[97] Hruska Z., Yao H., Kincaid R., Brown R.L., Bhatnagar D., Cleveland T.E. (2017). Temporal effects on internal fluorescence emissions associated with aflatoxin contamination from corn kernel cross-sections inoculated with toxigenic and atoxigenic *Aspergillus flavus*. Front. Microbiol..

[98] Hyde K.D., Al-Hatmi A.M.S., Andersen B., Boekhout T., Buzina W., Dawson T.L., Eastwood D.C., Jones E.B.G., de Hoog S., Kang Y. (2018). The world’s ten most feared fungi. Fungal Divers..

[99] Iizumi T. (2019). Emerging adaptation to climate change in agriculture. Adaptation to Climate Change in Agriculture: Research and Practices.

[100] Janić Hajnal E., Kos J., Krulj J., Krstović S., Jajić I., Pezo L., Šarić B., Nedeljković N. (2017). Aflatoxins contamination of maize in serbia: The impact of weather conditions in 2015. Food Addit. Contam. Part A Chem. Anal. Control Expo. Risk Assess..

[101] Jesmin R., Chanda A. (2020). Restricting mycotoxins without killing the producers: A new paradigm in nano-fungal interactions. Appl. Microbiol. Biotechnol..

[102] Kaminiaris M.D., Tsitsigiannis D.I. (2019). Pre-harvest management strategies to control aflatoxin contamination in crops. Aflatoxins: Biochemistry, Toxicology, Public Health, Policies and Modern Methods of Analysis.

[103] Kaynarca H.D., Hecer C., Ulusoy B. (2019). Mycotoxin hazard in meat and meat products. Ataturk Univ. Vet. Bilimleri Derg..

[104] Keriene I., Mankeviciene A., Cesnuleviciene R. (2018). Risk factors for mycotoxin contamination of buckwheat grain and its products. World Mycotoxin J..

[105] Klvana M., Bren U. (2019). Aflatoxin B1–Formamidopyrimidine DNA Adducts: Relationships between Structures, Free Energies, and Melting Temperatures. Molecules.

[106] Knutsen H.K., Alexander J., Barregard L., Bignami M., Brüschweiler B., Ceccatelli S., Cottrill B., DiNovi M., Edler L., Grasl-Kraupp B. (2018). Effect on public health of a possible increase of the maximum level for ‘aflatoxin total’ from 4 to 10 μg/kg in peanuts and processed products thereof, intended for direct human consumption or use as an ingredient in foodstuffs. EFSA J..

[107] Kovač T., Borišev I., Crevar B., Čačić Kenjerić F., Kovač M., Strelec I., Ezekiel C.N., Sulyok M., Krska R., Šarkanj B. (2018). Fullerol c_60(oh)24_ nanoparticles modulate aflatoxin b_1_ biosynthesis in *Aspergillus flavus*. Sci. Rep..

[108] Kovač T., Borišev I., Kovač M., Lončarić A., Čačić Kenjerić F., Djordjevic A., Strelec I., Ezekiel C.N., Sulyok M., Krska R. (2020). Impact of fullerol c_60(oh)24_ nanoparticles on the production of emerging toxins by *Aspergillus flavus*. Sci. Rep..

[109] Kovač T., Kovač M., Strelec I., Nevistić A., Molnar M. (2017). Antifungal and antiaflatoxigenic activities of coumarinyl thiosemicarbazides against *Aspergillus flavus* nrrl 3251. Arh. za Hig. Rada i Toksikol..

[110] Kovač T., Šarkanj B., Borišev I., Djordjevic A., Jović D., Lončarić A., Babić J., Jozinović A., Krska T., Gangl J. (2020). Fullerol c_60(oh)24_ nanoparticles affect secondary metabolite profile of important foodborne mycotoxigenic fungi in vitro. Toxins.

[111] Kovač T., Šarkanj B., Crevar B., Kovač M., Lončarić A., Strelec I., Ezekiel C.N., Sulyok M., Krska R. (2018). *Aspergillus flavus* nrrl 3251 growth, oxidative status, and aflatoxins production ability in vitro under different illumination regimes. Toxins.

[112] Kovač T., Šarkanj B., Klapec T., Borišev I., Kovač M., Nevistić A., Strelec I. (2018). Antiaflatoxigenic effect of fullerene c_60_ nanoparticles at environmentally plausible concentrations. AMB Express.

[113] Kövesi B., Cserháti M., Erdélyi M., Zándoki E., Mézes M., Balogh K. (2020). Lack of dose- and time-dependent effects of aflatoxin b1 on gene expression and enzymes associated with lipid peroxidation and the glutathione redox system in chicken. Toxins.

[114] Krska R., De Nijs M., McNerney O., Pichler M., Gilbert J., Edwards S., Suman M., Magan N., Rossi V., Van Der Fels-Klerx H. (2016). Safe food and feed through an integrated toolbox for mycotoxin management: The MyToolBox approach. World Mycotoxin J..

[115] Krulj J., Đisalov J., Bocarov-Stancic A., Pezo L., Kojic J., Vidaković A., Solarov M.B. (2018). Occurrence of aflatoxin B1 in Triticum species inoculated with *Aspergillus flavus*. World Mycotoxin J..

[116] Ksenija N. (2018). Mycotoxins—Climate impact and steps to prevention based on prediction. Acta Vet..

[117] Kumphanda J., Matumba L., Whitaker T., Kasapila W., Sandahl J. (2019). Maize meal slurry mixing: An economical recipe for precise aflatoxin quantitation. World Mycotoxin J..

[118] Labanca F., Raimondi A., Fontanelli M., Pisuttu C., Rallo G., Galli F., Conte G., Pellegrini E. (2019). The effects of climate change on livestock production systems: The cases of mycotoxins in animal feed and animal heat stress. Agrochimica.

[119] Lanubile A., Maschietto V., Battilani P., Marocco A. (2017). Infection with toxigenic and atoxigenic strains of *Aspergillus flavus* induces different transcriptional signatures in maize kernels. J. Plant Interact..

[120] Leong Y.H., Ahmad N.I., Awang R. (2017). Occurrence, human exposure and the current trends of exposure measurements for aflatoxins. Focus on Aflatoxins Research.

[121] Magan N., Medina Á. (2016). Integrating gene expression, ecology and mycotoxin production by *Fusarium* and *Aspergillus* species in relation to interacting environmental factors. World Mycotoxin J..

[122] Mahato D.K., Lee K.E., Kamle M., Devi S., Dewangan K.N., Kumar P., Kang S.G. (2019). Aflatoxins in Food and Feed: An Overview on Prevalence, Detection and Control Strategies. Front. Microbiol..

[123] Mangasuli S.N. (2020). Synthesis of novel Isatin-Dithiocarbamate hybrids: An approach to microwave and potent antimicrobial agents. Chem. Data Collect..

[124] Martins C., Vidal A., De Boevre M., De Saeger S., Nunes C., Torres D., Goios A., Lopes C., Alvito P., Assunção R. (2020). Burden of disease associated with dietary exposure to carcinogenic aflatoxins in portugal using human biomonitoring approach. Food Res. Int..

[125] Masiello M., Somma S., Ghionna V., Francesco Logrieco A., Moretti A. (2019). In vitro and in field response of different fungicides against *Aspergillus flavus* and *Fusarium* species causing ear rot disease of maize. Toxins.

[126] Masiello M., Somma S., Haidukowski M., Logrieco A.F., Moretti A. (2020). Genetic polymorphisms associated to sdhi fungicides resistance in selected *Aspergillus flavus* strains and relation with aflatoxin production. Int. J. Microbiol..

[127] Mauro A., Garcia-Cela E., Pietri A., Cotty P.J., Battilani P. (2018). Biological control products for aflatoxin prevention in italy: Commercial field evaluation of atoxigenic *Aspergillus flavus* active ingredients. Toxins.

[128] Medina A., Akbar A., Baazeem A., Rodriguez A., Magan N. (2017). Climate change, food security and mycotoxins: Do we know enough?. Fungal Biol. Rev..

[129] Mesterhazy A., Toth E.T.T., Szel S., Varga M., Toth B. (2020). Resistance of Maize Hybrids to *Fusarium graminearum*, *F. culmorum*, and *F. verticillioides* Ear Rots with Toothpick and Silk Channel Inoculation, as Well as Their Toxin Production. Agronomy.

[130] Michelmore R., Coaker G., Bart R., Beattie G., Bent A., Bruce T., Cameron D., Dangl J., Dinesh-Kumar S., Edwards R. (2017). Foundational and Translational Research Opportunities to Improve Plant Health. Mol. Plant-Microbe Interact..

[131] Moretti A., Pascale M., Logrieco A.F. (2019). Mycotoxin risks under a climate change scenario in Europe. Trends Food Sci. Technol..

[132] Mshelia L.P., Selamat J., Samsudin N.I.P., Rafii M.Y., Mutalib N.-A.A., Nordin N., Berthiller F. (2020). Effect of Temperature, Water Activity and Carbon Dioxide on Fungal Growth and Mycotoxin Production of Acclimatised Isolates of *Fusarium verticillioides* and *F. graminearum*. Toxins.

[133] Munkvold G.P., Arias S., Taschl I., Gruber-Dorninger C. (2018). Mycotoxins in corn: Occurrence, impacts, and management. Corn: Chemistry and Technology.

[134] Myndrul V., Coy E., Bechelany M., Iatsunskyi I. (2021). Photoluminescence label-free immunosensor for the detection of Aflatoxin B1 using polyacrylonitrile/zinc oxide nanofibers. Mater. Sci. Eng. C.

[135] Nabwire W.R., Ombaka J., Dick C.P., Strickland C., Tang L., Xue K.S., Wang J.-S. (2019). Aflatoxin in household maize for human consumption in Kenya, East Africa. Food Addit. Contam. Part B.

[136] Nazhand A., Durazzo A., Lucarini M., Souto E.B., Santini A. (2020). Characteristics, Occurrence, Detection and Detoxification of Aflatoxins in Foods and Feeds. Foods.

[137] Nogueira L.M., Yabroff K.R., Bernstein A. (2020). Climate change and cancer. CA Cancer J. Clin..

[138] Nugent A.P., Thielecke F. (2019). Wholegrains and health: Many benefits but do contaminants pose any risk?. Nutr. Bull..

[139] Nugraha A., Khotimah K., Rietjens I.M. (2018). Risk assessment of aflatoxin B1 exposure from maize and peanut consumption in Indonesia using the margin of exposure and liver cancer risk estimation approaches. Food Chem. Toxicol..

[140] Nurerk P., Bunkoed W., Kanatharana P., Bunkoed O. (2018). A miniaturized solid-phase extraction adsorbent of calix[4]arene-functionalized graphene oxide/polydopamine-coated cellulose acetate for the analysis of aflatoxins in corn. J. Sep. Sci..

[141] Oliveira M., Vasconcelos V. (2020). Occurrence of Mycotoxins in Fish Feed and Its Effects: A Review. Toxins.

[142] Ortega-Beltran A., Cotty P.J. (2018). Frequent shifts in *Aspergillus flavus* populations associated with maize production in sonora, mexico. Phytopathology.

[143] Palacios-Rojas N., McCulley L., Kaeppler M., Titcomb T.J., Gunaratna N.S., Lopez-Ridaura S., Tanumihardjo S.A. (2020). Mining maize diversity and improving its nutritional aspects within agro-food systems. Compr. Rev. Food Sci. Food Saf..

[144] Pascale M., Logrieco A.F., Graeber M., Hirschberger M., Reichel M., Lippolis V., De Girolamo A., Lattanzio V.M.T., Slettengren K. (2020). Aflatoxin reduction in maize by industrial-scale cleaning solutions. Toxins.

[145] Paterson R.R.M., Venâncio A., Lima N., Guilloux-Bénatier M., Rousseaux S. (2018). Predominant mycotoxins, mycotoxigenic fungi and climate change related to wine. Food Res. Int..

[146] Peles F., Sipos P., Győri Z., Pfliegler W.P., Giacometti F., Serraino A., Pagliuca G., Gazzotti T., Pócsi I. (2019). Adverse effects, transformation and channeling of aflatoxins into food raw materials in livestock. Front. Microbiol..

[147] Peña-Rodas O., Martinez-Lopez R., Hernandez-Rauda R. (2018). Occurrence of Aflatoxin M1 in cow milk in El Salvador: Results from a two-year survey. Toxicol. Rep..

[148] Peña-Rodas O., Martinez-Lopez R., Pineda-Rivas M., Hernandez-Rauda R. (2020). Aflatoxin M1 in Nicaraguan and locally made hard white cheeses marketed in El Salvador. Toxicol. Rep..

[149] Perczak A., Goliński P., Bryła M., Waśkiewicz A. (2018). The efficiency of lactic acid bacteria against pathogenic fungi and mycotoxins. Arh. za Hig. Rada i Toksikol..

[150] Pimpitak U., Rengpipat S., Phutong S., Buakeaw A., Komolpis K. (2020). Development and validation of a lateral flow immunoassay for the detection of aflatoxin m_1_ in raw and commercialised milks. Int. J. Dairy Technol..

[151] Pleadin J., Zadravec M., Lešić T., Frece J., Markov K., Vasilj V. (2020). Climate change—A potential threat for increasing occurrences of mycotoxins. Vet. Stanica.

[152] Ponce-García N., Serna-Saldivar S.O., Garcia-Lara S. (2018). Fumonisins and their analogues in contaminated corn and its processed foods—A review. Food Addit. Contam. Part A Chem. Anal. Control Expo. Risk Assess..

[153] Poór M., Bálint M., Hetényi C., Gődér B., Kunsági-Máté S., Kőszegi T., Lemli B. (2017). Investigation of non-covalent interactions of aflatoxins (b1, b2, g1, g2, and m1) with serum albumin. Toxins.

[154] Porto Y.D., Trombete F.M., Freitas-Silva O., de Castro I.M., Direito G.M., Ascheri J.L.R. (2019). Gaseous ozonation to reduce aflatoxins levels and microbial contamination in corn grits. Microorganisms.

[155] Qin M., Liang J., Yang D., Yang X., Cao P., Wang X., Ma N., Zhang L. (2021). Spatial analysis of dietary exposure of aflatoxins in peanuts and peanut oil in different areas of China. Food Res. Int..

[156] Ráduly Z., Szabó L., Madar A., Pócsi I., Csernoch L. (2020). Toxicological and medical aspects of *Aspergillus*-derived mycotoxins entering the feed and food chain. Front. Microbiol..

[157] Raiten D.J., Aimone A.M. (2017). The intersection of climate/environment, food, nutrition and health: Crisis and opportunity. Curr. Opin. Biotechnol..

[158] Renaud J.B., Miller J.D., Sumarah M.W. (2019). Mycotoxin testing paradigm: Challenges and opportunities for the future. J. AOAC Int..

[159] Ricciardi W., Marcheggiani S., Puccinelli C., Carere M., Sofia T., Giuliano F., Dogliotti E., Mancini L., Agrimi U., Alleva E. (2019). Health and climate change: Science calls for global action. Ann. dell’Istituto Super. di Sanita.

[160] Righetti L., Paglia G., Galaverna G., Dall’Asta C. (2016). Recent advances and future challenges in modified mycotoxin analysis: Why hrms has become a key instrument in food contaminant research. Toxins.

[161] Rushing B.R., Selim M.I. (2018). Adduction to arginine detoxifies aflatoxin b_1_ by eliminating genotoxicity and altering in vitro toxicokinetic profiles. Oncotarget.

[162] Rushing B.R., Selim M.I. (2019). Aflatoxin b_1_: A review on metabolism, toxicity, occurrence in food, occupational exposure, and detoxification methods. Food Chem. Toxicol..

[163] Šarkanj B., Ezekiel C.N., Turner P.C., Abia W.A., Rychlik M., Krska R., Sulyok M., Warth B. (2018). Ultra-sensitive, stable isotope assisted quantification of multiple urinary mycotoxin exposure biomarkers. Anal. Chim. Acta.

[164] Sarrocco S., Mauro A., Battilani P. (2019). Use of competitive filamentous fungi as an alternative approach for mycotoxin risk reduction in staple cereals: State of art and future perspectives. Toxins.

[165] Satterlee T., Cary J.W., Calvo A.M. (2016). Rmta, a putative arginine methyltransferase, regulates secondary metabolism and development in *Aspergillus flavus*. PLoS ONE.

[166] Savić Z., Dudaš T., Loc M., Grahovac M., Budakov D., Jajić I., Krstović S., Barošević T., Krska R., Sulyok M. (2020). Biological control of aflatoxin in maize grown in serbia. Toxins.

[167] Schaarschmidt S., Fauhl-Hassek C. (2021). The fate of mycotoxins during secondary food processing of maize for human consumption. Compr. Rev. Food Sci. Food Saf..

[168] Schaarschmidt S., Fauhl-Hassek C. (2021). The fate of mycotoxins during the primary food processing of maize. Food Control.

[169] Schrenk D., Bignami M., Bodin L., Chipman J.K., del Mazo J., Grasl-Kraupp B., Hogstrand C., Hoogenboom L., Leblanc J.C., Nebbia C.S. (2020). Risk assessment of aflatoxins in food. EFSA J..

[170] Singh P., Callicott K.A., Orbach M.J., Cotty P.J. (2020). Molecular Analysis of S-morphology Aflatoxin Producers from the United States Reveals Previously Unknown Diversity and Two New Taxa. Front. Microbiol..

[171] Smith J.W., Groopman J.D. (2018). Aflatoxins. Encyclopedia of Cancer.

[172] Soares R.R.G., Ricelli A., Fanelli C., Caputo D., De Cesare G., Chu V., Aires-Barros M.R., Conde J.P. (2018). Advances, challenges and opportunities for point-of-need screening of mycotoxins in foods and feeds. Analyst.

[173] Sojinrin T., Liu K., Wang K., Cui D., Byrne H.J., Curtin J.F., Tian F. (2019). Developing Gold Nanoparticles-Conjugated Aflatoxin B1 Antifungal Strips. Int. J. Mol. Sci..

[174] Söylemez T., Yamaç M., Yıldız Z. (2020). Statistical optimization of cultural variables for enzymatic degradation of aflatoxin b_1_ by *Panus neostrigosus*. Toxicon.

[175] Steiner D., Sulyok M., Malachová A., Mueller A., Krska R. (2020). Realizing the simultaneous liquid chromatography-tandem mass spectrometry based quantification of >1200 biotoxins, pesticides and veterinary drugs in complex feed. J. Chromatogr. A.

[176] Stepman F. (2018). Scaling-up the impact of aflatoxin research in africa. The role of social sciences. Toxins.

[177] Sun Y., Liu Z., Liu D., Chen J., Gan F., Huang K. (2018). Low-Level Aflatoxin B1 Promotes Influenza Infection and Modulates a Switch in Macrophage Polarization from M1 to M2. Cell. Physiol. Biochem..

[178] Sun Y., Su J., Liu Z., Liu D., Gan F., Chen X., Huang K. (2018). Aflatoxin b_1_ promotes influenza replication and increases virus related lung damage via activation of tlr4 signaling. Front. Immunol..

[179] Sun Y., Su J., Yang S., Liu Z., Liu D., Gan F., Chen X., Huang K. (2019). Mannan oligosaccharide protects against the aflatoxin-b1-promoted influenza replication and tissue damages in a toll-like-receptor-4-dependent manner. J. Agric. Food Chem..

[180] Szabo B., Toth B., Toldine E.T., Varga M., Kovacs N., Varga J., Kocsube S., Palagyi A., Bagi F., Budakov D. (2018). A new concept to secure food safety standards against *Fusarium* species and *Aspergillus flavus* and their toxins in maize. Toxins.

[181] Tacconi C., Cucina M., Pezzolla D., Zadra C., Gigliotti G. (2018). Effect of the mycotoxin aflatoxin b_1_ on a semi-continuous anaerobic digestion process. Waste Manag..

[182] Thielecke F., Nugent A.P. (2018). Contaminants in grain—A major risk for whole grain safety?. Nutrients.

[183] Toreti A., Bassu S., Ceglar A., Zampieri M. (2018). Climate change and crop yields. Encyclopedia of Food Security and Sustainability.

[184] Udovicki B., Audenaert K., De Saeger S., Rajkovic A. (2018). Overview on the mycotoxins incidence in serbia in the period 2004–2016. Toxins.

[185] Udovicki B., Djekic I., Gajdos Kljusuric J., Papageorgiou M., Skendi A., Djugum J., Rajkovic A. (2019). Exposure assessment and risk characterization of aflatoxins intake through consumption of maize products in the adult populations of Serbia, Croatia and Greece. Food Addit. Contam. Part A Chem. Anal. Control Expo. Risk Assess..

[186] Udovicki B., Djekic I., Stankovic S., Obradovic A., Rajkovic A. (2019). Impact of climatic conditions on fumonisins in maize grown in Serbia. World Mycotoxin J..

[187] Uka V., Cary J.W., Lebar M.D., Puel O., De Saeger S., Diana Di Mavungu J. (2020). Chemical repertoire and biosynthetic machinery of the *Aspergillus flavus* secondary metabolome: A review. Compr. Rev. Food Sci. Food Saf..

[188] Valencia-Quintana R., Milić M., Jakšić D., Klarić M.Š., Tenorio-Arvide M.G., Pérez-Flores G.A., Bonassi S., Sánchez-Alarcón J. (2020). Environment changes, aflatoxins, and health issues, a review. Int. J. Environ. Res. Public Health.

[189] van der Fels-Klerx H.J., Camenzuli L. (2016). Effects of milk yield, feed composition, and feed contamination with aflatoxin b_1_ on the aflatoxin m_1_ concentration in dairy cows’ milk investigated using monte carlo simulation modelling. Toxins.

[190] Van Der Fels-Klerx H.J., Liu C., Battilani P. (2016). Modelling climate change impacts on mycotoxin contamination. World Mycotoxin J..

[191] Van der Fels-Klerx H.J., Vermeulen L.C., Gavai A.K., Liu C. (2019). Climate change impacts on aflatoxin b_1_ in maize and aflatoxin m_1_ in milk: A case study of maize grown in eastern europe and imported to the netherlands. PLoS ONE.

[192] Vandicke J., De Visschere K., Croubels S., De Saeger S., Audenaert K., Haesaert G. (2019). Mycotoxins in flanders’ fields: Occurrence and correlations with *Fusarium* species in whole-plant harvested maize. Microorganisms.

[193] Verheecke C., Liboz T., Mathieu F. (2016). Microbial degradation of aflatoxin b1: Current status and future advances. Int. J. Food Microbiol..

[194] Viegas S., Assunção R., Martins C., Nunes C., Osteresch B., Twarużek M., Kosicki R., Grajewski J., Ribeiro E., Viegas C. (2019). Occupational exposure to mycotoxins in swine production: Environmental and biological monitoring approaches. Toxins.

[195] Viegas S., Assunção R., Nunes C., Osteresch B., Twarużek M., Kosicki R., Grajewski J., Martins C., Alvito P., Almeida A. (2018). Exposure assessment to mycotoxins in a portuguese fresh bread dough company by using a multi-biomarker approach. Toxins.

[196] Viegas S., Assunção R., Twarużek M., Kosicki R., Grajewski J., Viegas C. (2020). Mycotoxins feed contamination in a dairy farm—Potential implications for milk contamination and workers’ exposure in a one health approach. J. Sci. Food Agric..

[197] Wacoo A.P., Wendiro D., Nanyonga S., Hawumba J.F., Sybesma W., Kort R. (2018). Feasibility of a novel on-site detection method for aflatoxin in maize flour from markets and selected households in kampala, uganda. Toxins.

[198] Windham G.L., Williams W.P., Mylroie J.E., Reid C.X., Womack E.D. (2018). A histological study of *Aspergillus flavus* colonization of wound inoculated maize kernels of resistant and susceptible maize hybrids in the field. Front. Microbiol..

[199] Winter G., Pereg L. (2019). A review on the relation between soil and mycotoxins: Effect of aflatoxin on field, food and finance. Eur. J. Soil Sci.

[200] Yu J., Hennessy D.A., Wu F. (2020). The impact of bt corn on aflatoxin-related insurance claims in the united states. Sci. Rep..

[201] Zhao X., Wei J., Zhou Y., Kong W., Yang M. (2017). Quality evaluation of *alpinia oxyphylla* after *Aspergillus flavus* infection for storage conditions optimization. AMB Express.

[202] Zhou X., Gan F., Hou L., Liu Z., Su J., Lin Z., Le G., Huang K. (2019). Aflatoxin b1 induces immunotoxicity through the DNA methyltransferase-mediated jak2/stat3 pathway in 3d4/21 cells. J. Agric. Food Chem..

[203] (2015). Review of the Draft Interagency Report on the Impacts of Climate Change on Human Health in the United States.

[204] Battilani P., Camardo Leggieri M. (2015). Predictive modelling of aflatoxin contamination to support maize chain management. World Mycotoxin J..

[205] Clark G.C., Casewell N.R., Elliott C.T., Harvey A.L., Jamieson A.G., Strong P.N., Turner A.D. (2019). Friends or foes? Emerging impacts of biological toxins. Trends Biochem. Sci..

[206] Csáki K.F., Szabó M.S., Túri M.S. (2014). Possibilities for the decrease of aflatoxin contamination in food chain. Elelmvizsg. Kozl..

[207] De Nijs M., Mengelers M.J.B., Boon P.E., Heyndrickx E., Hoogenboom L.A.P., Lopez P., Mol H.G.J. (2016). Strategies for estimating human exposure to mycotoxins via food. World Mycotoxin J..

[208] Donohoe T., Garnett K., Lansink A.O., Afonso A., Noteborn H. (2018). Emerging risks identification on food and feed—Efsa. EFSA J..

[209] Eskola M., Altieri A., Galobart J. (2018). Overview of the activities of the european food safety authority on mycotoxins in food and feed. World Mycotoxin J..

[210] Gilbert M.K., Mack B.M., Payne G.A., Bhatnagar D. (2016). Use of functional genomics to assess the climate change impact on *Aspergillus flavus* and aflatoxin production. World Mycotoxin J..

[211] Giorni P., Camardo Leggieri M., Magan N., Battilani P. (2012). Comparison of temperature and moisture requirements for sporulation of *Aspergillus flavus* sclerotia on natural and artificial substrates. Fungal Biol..

[212] Gómez J.V., Tarazona A., Mateo F., Jiménez M., Mateo E.M. (2019). Potential impact of engineered silver nanoparticles in the control of aflatoxins, ochratoxin a and the main aflatoxigenic and ochratoxigenic species affecting foods. Food Control.

[213] Gruber-Dorninger C., Jenkins T., Schatzmayr G. (2019). Global mycotoxin occurrence in feed: A ten-year survey. Toxins.

[214] Hernández-Martínez R., Navarro-Blasco I. (2015). Surveillance of aflatoxin content in dairy cow feedstuff from navarra (Spain). Anim. Feed Sci. Technol..

[215] Kanapitsas A., Batrinou A., Aravantinos A., Sflomos C., Markaki P. (2016). Gamma radiation inhibits the production of ochratoxin a by *Aspergillus carbonarius*. Development of a method for ota determination in raisins. Food Biosci..

[216] Kovač T., Šarkanj B., Klapec T., Borišev I., Kovač M., Nevistić A., Strelec I. (2017). Fullerol c_60(oh)24_ nanoparticles and mycotoxigenic fungi: A preliminary investigation into modulation of mycotoxin production. Environ. Sci. Pollut. Res..

[217] Lagogianni C.S., Tsitsigiannis D.I. (2018). Effective chemical management for prevention of aflatoxins in maize. Phytopathol. Mediterr..

[218] Lagogianni C.S., Tsitsigiannis D.I. (2019). Effective biopesticides and biostimulants to reduce aflatoxins in maize fields. Front. Microbiol..

[219] Lulamba T.E., Stafford R.A., Njobeh P.B. (2019). A sub-saharan african perspective on mycotoxins in beer—A review. J. Inst. Brew..

[220] Magan N., Medina A. (2016). Mycotoxins, food security and climate change: Do we know enough?. Microbiol. Today.

[221] Malissiova E., Manouras A. (2017). Monitoring aflatoxin m_1_ levels in donkey milk produced in greece, intended for human consumption. World Mycotoxin J..

[222] Malissiova E., Tsakalof A., Arvanitoyannis I.S., Katsafliaka A., Katsioulis A., Tserkezou P., Koureas M., Govaris A., Hadjichristodoulou C. (2013). Monitoring aflatoxin m_1_ levels in ewe’s and goat’s milk in thessaly, greece; potential risk factors under organic and conventional production schemes. Food Control.

[223] Manouras A., Malissiova E. (2015). Occurrence of aflatoxins in compound feeds and feed materials for dairy livestock in central greece. Hell. Veter- Med Soc..

[224] Mateo E.M., Gómez J.V., Gimeno-Adelantado J.V., Romera D., Mateo-Castro R., Jiménez M. (2017). Assessment of azole fungicides as a tool to control growth of *Aspergillus flavus* and aflatoxin b_1_ and b_2_ production in maize. Food Addit. Contam. Part A Chem. Anal. Control Expo. Risk Assess..

[225] Matumba L., Sulyok M., Monjerezi M., Biswick T., Krska R. (2015). Fungal metabolites diversity in maize and associated human dietary exposures relate to micro-climatic patterns in malawi. World Mycotoxin J..

[226] Medina A., Rodriguez A., Magan N., Botana M.J., Sainz L.M. (2015). Changes in environmental factors driven by climate change: Effects on the ecophysiology of mycotoxigenic fungi. Climate Change and Mycotoxins.

[227] Medina Á., Rodríguez A., Magan N. (2015). Climate change and mycotoxigenic fungi: Impacts on mycotoxin production. Curr. Opin. Food Sci..

[228] Moretti A., Logrieco A.F., Botana M.J., Sainz L.M. (2015). Climate change effects on the biodiversity of mycotoxigenic fungi and their mycotoxins in preharvest conditions in europe. Climate Change and Mycotoxins.

[229] Nazari L., Manstretta V., Rossi V. (2016). A non-linear model for temperature-dependent sporulation and t-2 and ht-2 production of *Fusarium langsethiae* and *Fusarium sporotrichioides*. Fungal Biol..

[230] Pangga I.B., Hanan J., Chakraborty S. (2013). Climate change impacts on plant canopy architecture: Implications for pest and pathogen management. European J. Plant Pathol..

[231] Pangga I.B., Salvacion A.R., Cumagun C.J.R., Botana M.J., Sainz L.M. (2015). Climate change and plant diseases caused by mycotoxigenic fungi: Implications for food security. Climate Change and Mycotoxins.

[232] Paris M.P.K., Liu Y.J., Nahrer K., Binder E.M., Botana M.J., Sainz L.M. (2015). Climate change impacts on mycotoxin production. Climate Change and Mycotoxins.

[233] Pautasso M., Petter F., Rortais A., Roy A.S. (2015). Emerging risks to plant health: A european perspective. CAB Reviews: Perspect. Agric. Vet. Sci. Nutr. Nat. Resour..

[234] Pecorelli I., Guarducci N., Von Holst C., Bibi R., Pascale M., Ciasca B., Logrieco A.F., Lattanzio V.M.T. (2020). Critical comparison of analytical performances of two immunoassay methods for rapid detection of aflatoxin m_1_ in milk. Toxins.

[235] Robinson T., Altieri A., Chiusolo A., Dorne J.L., Goumperis T., Rortais A., Deluyker H., Silano V., Liem D. (2012). Efsa’s approach to identifying emerging risks in food and feed: Taking stock and looking forward. EFSA J..

[236] Rodríguez-Blanco M., Ramos A.J., Prim M., Sanchis V., Marín S. (2020). Usefulness of the analytical control of aflatoxins in feedstuffs for dairy cows for the prevention of aflatoxin m_1_ in milk. Mycotoxin Res..

[237] Sainz M.J., Alfonso A., Botana L.M. (2015). Considerations about international mycotoxin legislation, food security, and climate change. Climate Change and Mycotoxins.

[238] Sckokai P., Veneziani M., Moro D., Castellari E. (2014). Consumer willingness to pay for food safety: The case of mycotoxins in milk. Bio-Based Appl. Econ..

[239] Torović L. (2015). Aflatoxin m_1_ in processed milk and infant formulae and corresponding exposure of adult population in serbia in 2013–2014. Food Addit. Contam. Part B Surveill..

[240] Trevisani M., Farkas Z., Serraino A., Zambrini A.V., Pizzamiglio V., Giacometti F., Ambrus Á. (2014). Analysis of industry-generated data. Part 1: A baseline for the development of a tool to assist the milk industry in designing sampling plans for controlling aflatoxin M_1_ in milk. Food Addit. Contam. Part A Chem. Anal. Control Expo. Risk Assess..

[241] Van de Perre E., Jacxsens L., Liu C., Devlieghere F., De Meulenaer B. (2015). Climate impact on *alternaria* moulds and their mycotoxins in fresh produce: The case of the tomato chain. Food Res. Int..

[242] Van der Fels-Klerx H.J., Olesen J.E., Madsen M.S., Goedhart P.W. (2012). Climate change increases deoxynivalenol contamination of wheat in north-western europe. Food Addit. Contam. Part A Chem. Anal. Control Expo. Risk Assess..

[243] Drasticdata Tool. https://www.drasticdata.nl/index.htm.

[244] Dobolyi C., Sebok F., Varga J., Kocsube S., Szigeti G., Baranyi N., Szecsi A., Toth B., Varga M., Kriszt B. (2013). Occurrence of aflatoxin producing *Aspergillus flavus* isolates in maize kernel in hungary. Acta Aliment. (Budapest).

[245] Levic J., Gosic-Dondo S., Ivanovic D., Stankovic S., Krnjaja V., Bocarov-Stancic A., Stepanic A. (2013). An outbreak of *Aspergillus* species in response to environmental conditions in Serbia. Pestic. i Fitomedicina.

[246] Giorni P., Magan N., Pietri A., Bertuzzi T., Battilani P. (2007). Studies on *Aspergillus* section *flavi* isolated in northern Italy from maize. Int. J. Food Microbiol..

[247] Mauro A., Battilani P., Cotty P.J. (2015). Atoxigenic *Aspergillus flavus* endemic to italy for biocontrol of aflatoxins in maize. BioControl.

[248] Warnatzsch E.A., Reay D.S., Camardo Leggieri M., Battilani P. (2020). Climate change impact on aflatoxin contamination risk in malawi’s maize crops. Front. Sustain. Food Syst..

[249] Giorni P., Bertuzzi T., Battilani P. (2019). Impact of fungi co-occurrence on mycotoxin contamination in maize during the growing season. Front. Microbiol..

[250] Palumbo R., Goncalves A., Gkrillas A., Logrieco A., Dorne J.L., Dall’Asta C., Venancio A., Battilani P. (2020). Mycotoxins in maize: Mitigation actions, with a chain management approach. Phytopathol. Mediterr..

[251] Marín S., Freire L., Femenias A., Sant’Ana A.S. (2021). Use of predictive modelling as tool for prevention of fungal spoilage at different points of the food chain. Curr. Opin. Food Sci..

[252] Miedaner T., Juroszek P. (2021). Global warming and increasing maize cultivation demand comprehensive efforts in disease and insect resistance breeding in north-western europe. Plant Pathol..

[253] Fanzo J., Bellows A.L., Spiker M.L., Thorne-Lyman A.L., Bloem M.W. (2021). The importance of food systems and the environment for nutrition. Am. J. Clin. Nutr..

